# Excessive psychological stress preceding the onset of idiopathic cervical dystonia

**DOI:** 10.1007/s00702-023-02694-7

**Published:** 2023-09-29

**Authors:** Dirk Dressler, Bruno Kopp, Lizhen Pan, Fereshte Adib Saberi

**Affiliations:** 1 Movement Disorders Section, Department of Neurology, Hannover Medical School, Carl-Neuberg-Str. 1, 30625 Hannover, Germany; 2 Department of Neurology, Hannover Medical School, Hannover, Germany; 3 Department of Neurology, Neurotoxin Research Center, Tongji University School of Medicine, Shanghai, China; 4 IAB-Interdisciplinary Working Group for Movement Disorders, Hamburg, Germany

**Keywords:** Cervical dystonia, Idiopathic, Excessive psychological stress, Time course remission, Triggering mechanism, Pathophysiological elements

## Abstract

Idiopathic cervical dystonia (ICD) is the largest subgroup of dystonia. Psychological stress as a triggering factor has long been discussed, but detailed descriptions are lacking. We report on a group of 13 patients with ICD and preceding excessive psychological stress (age at ICD onset 39.0 ± 13.9 years, 7 females, 6 males). The observation period was 7.8 ± 5.0 years. Excessive psychological stress included partner conflicts (divorce and separation, domestic violence), special familial burdens, legal disputes and migration. It started 8.3 ± 3.9 months before ICD onset. In 85% of our patients (typical cases), ICD developed within 5.8 ± 4.4 weeks, then lasted 18.5 ± 8.3 months, before it started to remit 2.7 ± 0.8 years after its onset to 54.5 ± 35.3% of its maximal severity. Idiopathic dystonia is thought to be based upon a genetic predisposition triggered by epigenetic factors. Our study suggests that excessive psychological stress could be one of them. Pathophysiologic elements are only vaguely identified, but could include the endoplasmic reticulum stress response, cerebellar 5HT-2A receptors and the metabolism of heat shock proteins. Whilst the clinical presentation of ICD preceded by excessive psychological stress is typical, its course is atypical with rapid onset and fast and substantial remission.

## Introduction

Idiopathic dystonia is defined as dystonia occurring in the absence of identifiable inherited or acquired causes and in the absence of a psychogenic aetiology (Albanese et al. 2013). Idiopathic cervical dystonia (ICD) dystonia is the largest subgroup of dystonia (Dressler et al. 2022). Interactions between dystonia and psychological stress have long been described, either as an aggravating factor modulating the severity of existing dystonia, or as a trigger factor starting its onset. To our knowledge, larger case series of psychological stress as a triggering factor are surprisingly lacking. We wanted to provide a detailed description of a series of patients in whom ICD was preceded by excessive psychological stress.

## Methods

### Design

This study is a descriptive study in the form of a case collection series. Patients were followed up continuously in the Movement Disorders Section of Hannover Medical School for botulinum toxin therapy.

### Patients

Inclusion criteria consisted of (1) diagnosis of ICD; (2) excessive psychological stress during 6 months before ICD onset. Exclusion criteria included (1) cervical dystonia with inherited or acquired causes; (2) cervical dystonia with psychogenic aetiology; (3) non-dystonic torticollis.

### Study parameters

**Fig. 1:**
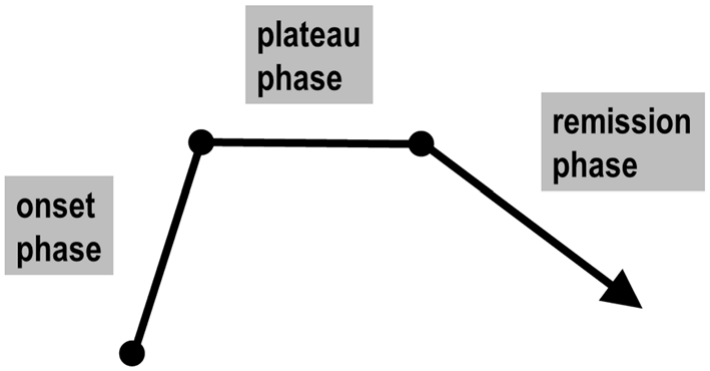
Schematic course of idiopathic cervical dystonia

Patient demographics described patient sex and patient age at ICD onset. The course of ICD consists of the onset phase, the plateau phase and the remission phase, as shown in Fig. 1. The observation period was defined as the time between ICD onset and last contact with the patient. The patient's family history of dystonia was derived from personal examination of family members, diagnoses by other physicians and signs and symptoms provided by the patient. The level of diagnostic accuracy may best be described as 'probable'. Exceptional psychological stress was defined as psychological stress of a severity the patient had never experienced before or thereafter.

### Ethical approval

The study was performed under the regulations of the local ethical committee of Hannover Medical School.

## Results

### Patients

Altogether, 13 patients with ICD and excessive psychological stress were identified and consecutively included in this study. Seven of them were female and six male. Their age at ICD onset was 39.0 ± 13.9 years. Three had a family history of dystonia. Nine patients were followed up throughout the entire course of their disease by one of the authors (DD). Four patients were initially seen elsewhere, before they joined our service. The observation period was 7.8 ± 5.0 years.

### Exceptional psychological stress

Table 1 gives the details of the exceptional psychological stress for each patient. Five patients experienced divorce or separation, four special caretaking burdens, four criminal charges or court litigations, three experienced migration and two domestic violence. Four patients were bearing psychiatric diagnoses including schizoaffective episode, anxiety disorder, sociophobia, depression and irritable personality disorder. Exceptional psychological stress started 8.3 ± 3.9 months before ICD onset and was present at ICD onset.

**Table 1:** Details of exceptional psychological stress in all cervical dystonia patients, where it was observed

Emigration from Afghanistan, forced marriage, victim of repeated domestic violence
Emigration from Iraq, alcoholism, divorce, job loss, loss of driver's licence, perpetrator of domestic violence
Job loss, divorce, loss of legal custody for two children, victim of domestic violence, refugee in women's shelter
Divorce, court litigation on legal custody for two children
Bankruptcy, loss of business, divorce, divorce litigation
Immigration from Kazakhstan, bankruptcy of employer, job loss, alcoholism
Anxiety disorder, six previous dystonia episodes, embezzlement charges, father with heart transplantation, change of jobs, neighbourhood dispute, intercollegiate dispute, severe disease of mother, mamma carcinoma diagnosis; duration of dystonia episodes: 4–8 months; first episode at age 23
Irritable personality, alcoholism, several convictions for violent crimes, conscription into the military service
Two disabled children, rejected by family, sudden obligation to take care of mother and parental home
In vitro fertilization, episode of schizoaffective psychosis with hospitalization
Sociophobia, obsessive–compulsive disorder, depression, long-term psychotherapy, abrupt start as trainee in big international corporation
Severe lovesickness
Divorce, moving address

### ICD

The clinical presentation of ICD in our cohort was indistinguishable from ICD not preceded by excessive psychological stress.

**Fig. 2:**
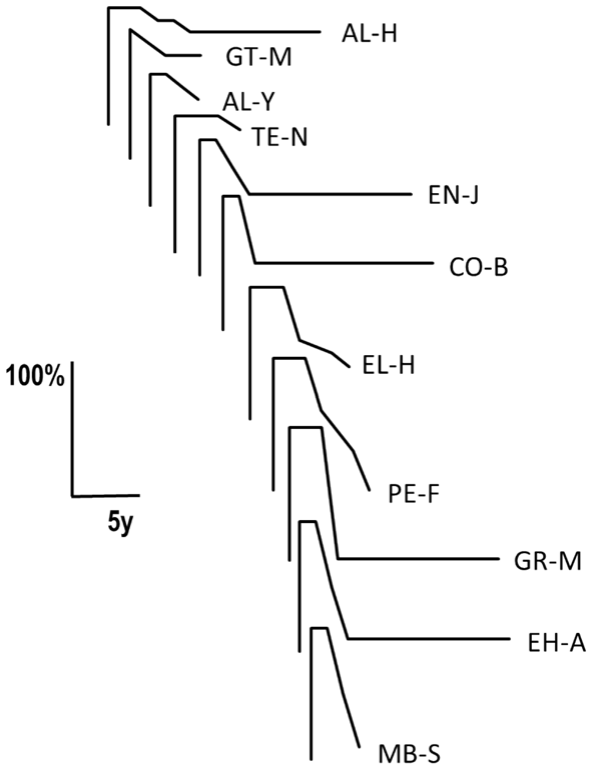
The course of idiopathic cervical dystonia with preceding excessive psychological stress (typical cases): individual patients

**Fig. 3:**
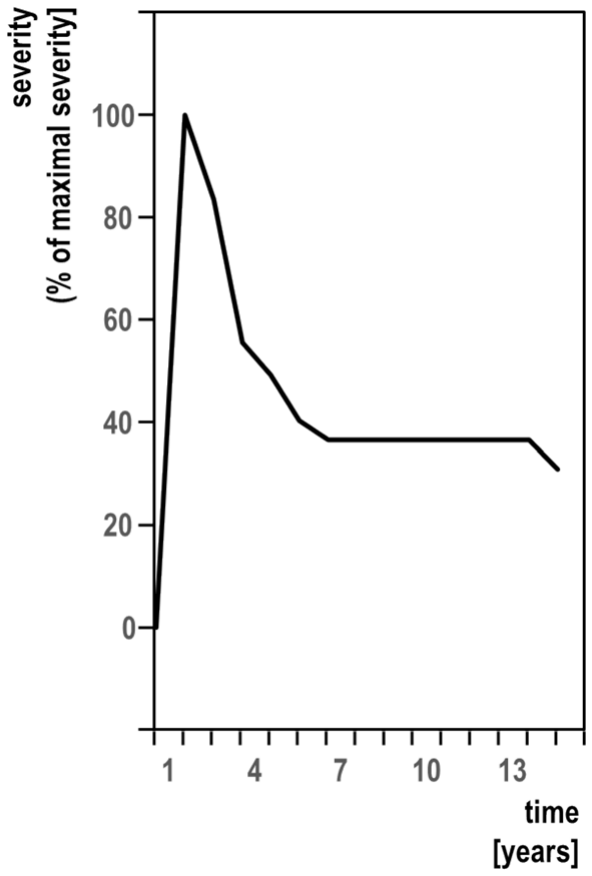
The course of idiopathic cervical dystonia with preceding excessive psychological stress (typical cases). Combined data

The numeric evaluation is shown in Table 2. In 11 out of 13 patients (85%) (typical cases), ICD developed within 5.8 ± 4.4 weeks, then lasted during the plateau phase for 18.5 ± 8.3 months, before it started to remit 2.7 ± 0.8 years after its onset to 54.5 ± 35.3% of its maximal severity. The course of ICD is shown for each typical patient in Fig. 2 and for the entire group of typical patients in Fig. 3. Two out of 13 patients (15%) (atypical cases) did not show remission over periods of 168 weeks and 48 weeks. One of them (MO-N) also had an atypically long progression phase of 156 weeks. One patient (GT-M) had six previous episodes of ICD lasting 4–6 months and first starting at age 23 years. All of the previous episodes occurred under some psychological stress, although not to the degree of the last one. Eight of our patients were seen immediately after the ICD onset. In four of them, ICD severity seemed more severe than in ICD without preceding psychological stress.

**Table 2:** The course of idiopathic cervical dystonia with preceding excessive psychological stress

	Typical cases 11/13 (85%)	Atypical cases 2/13 (15%)
Onset phase	Duration 5.8 ± 4.4 weeks	156 weeks (*n* = 1)
Plateau phase	Duration 18.5 ± 8.3 months	n/a
Remission phase	Duration 1.6 ± 0.7 years extent 54.5 ± 35.3%	No remission *(n* = 2)

*n/a* not applicable

## Discussion

### General observations

This is—to our knowledge—the only detailed study describing ICD with preceding excessive psychological stress. With 13 patients included, an observation period of 7.8 ± 5.0 years and personal long-term follow-ups by a single observer in most of the patients, it should provide robust data. Although most of the patients were followed prospectively, in four patients some data had to be retrieved retrospectively. Ideally, prospective designs should be favoured, but, due to the relative rarity of ICD, this can be difficult.

### Patients

CD patients reported here have an age of ICD onset of 39.0 ± 13.9 years. This seems young for an ICD patient population usually running in their 50s or 60s. However, here we documented age at ICD onset and not age at study inclusion as often used. Compared to an unpublished control group of ICD patients collected in our previous epidemiology study (Dressler et al. 2022), age at ICD onset was 46.9 ± 13.4 years, being similar to the age of ICD onset reported here. The usual 2:1 female preponderance of cervical ICD (Dressler et al. 2022) was not found in our study. Instead, we saw a female preponderance of 1.2 to 1, only. This might be attributed to the limited sample size. Four out of 13 patients (30%) were carrying psychiatric diagnoses including schizophrenia, affective disorders, anxiety and personality disorders.

### Exceptional psychological stress

Patients collected here experienced exceptional psychological stress never experienced before or thereafter. Obviously, defining psychological stress and attempts to quantify it are problematic, since psychological stress and its perception are highly subjective. We chose 'uniqueness' as a practical severity marker. Future studies might try to apply objective psychological stress measurement instruments. Exceptional psychological stress was generated by various typical conflict fields including partner conflicts (divorce and separation, domestic violence), special familial burdens, legal disputes and migration.

### ICD after excessive psychological stress

ICD started 8.3 ± 3.9 months after excessive psychological stress onset. In 85% of patients (typical cases), ICD developed within 5.8 ± 4.4 weeks, then lasted 18.5 ± 8.5 months, before it started to remit 2.7 ± 0.8 years after its onset to 54.5 ± 35.3% of its maximal severity. This course is entirely different from ICD not preceded by excessive psychological stress, where development of the full symptomatology usually takes several years and remissions are extremely rare and mostly minimal. Additionally, despite the lack of objective ICD severity scores, we felt, that overall severity of some of our patients was comparatively high.

### Relationship between excessive psychological stress and ICD

Our study shows a temporal relationship between excessive psychological stress and ICD. This could be interpreted as a mere coincidence. However, rarity of both phenomena and occurrence within a relatively short period of time point towards a causal relationship. Two patients were atypical. Patient GT-M is interesting in this respect: she experienced altogether six previous episodes of ICD lasting several months each, before the experienced the current lasting episode of ICD. Less intense psychological stress previously was related to shorter ICD episodes, whilst the current excessive psychological stress was related to a lasting ICD episode with reduced remission. Patient MO-N was also interesting. He was atypical with a long onset phase, a lack of remission and 'excessive psychological stress' in the form of lovesickness only. It might be speculated that in this case, psychological stress may have been a weak triggering factor.

### Underlying mechanisms

Idiopathic dystonia is thought to be based upon autosomal dominant inheritance with a markedly reduced penetrance of 12–15% (Waddy et al. 1991; Defazio et al. 1993). In ICD, unpublished data from a recent epidemiological study indicate a familial pattern in 20.5% of cases (Dressler et al. 2022). Low penetrance suggests an influence of epigenetic factors triggering or protecting against the hereditary trait. Possible triggering factors have included various forms of trauma, scoliosis, birth complications, childhood or adult infections and repetitive motor activity especially in task-specific dystonia (Jankovic et al. 1991; Martino et al. 2013; Schweinfurth et al. 2002; Defazio et al. 2003; Byl 2007). Psychological stress was suspected as a triggering for a long time. Anecdotal reports—often not published—circulated in medical circles, mainly on the association of life events and the onset of dystonia. However, to our knowledge, no detailed study has been performed on this association so far.

Pathophysiologic elements connecting excessive psychological stress and the onset of idiopathic dystonia have been only vaguely identified, but could include the torsinA mediated endoplasmic reticulum stress response (Chen et al. 2021) and cerebellar 5HT-2A receptors (Kim et al. 2021). The notion that DYT 1 gene alterations interfere with the metabolism of heat shock proteins could also point towards a potential pathophysiological mechanism (Ozelius et al. 1998). The high prevalence of premorbid psychiatric conditions in our cohort could also point to the role of suboptimal psychological stress coping mechanisms generating a special vulnerability of the central nervous system against psychological stress.

### Outlook

Psychological stress can interfere with dystonia in various ways. It can cause psychogenic dystonia and aggravate existing dystonia. Our study suggests that psychological stress might also trigger the onset of—genetically preformed—idiopathic dystonia. In the light of our current findings, war trauma-associated dystonia frequently reported after World War I should be re-evaluated as to whether it represents idiopathic dystonia triggered by excessive psychological stress rather than psychogenic dystonia.

Further studies of our group are currently underway to compare the natural course of idiopathic cervical dystonia preceded by excessive psychological stress with idiopathic cervical dystonia not preceded by excessive psychological stress in a direct head-to head comparison design.

Basic science data, especially those coming from genetics, are desperately needed to provide further insight into mechanisms linking stress and dystonia. Understanding mechanisms linking excessive psychological stress and dystonia might also provide insight into how milder forms of stress modulates dystonia and how eventually, manipulations of these mechanisms might be used for therapeutic purposes.
